# [Corrigendum] Ophiopogonin B suppresses the metastasis and angiogenesis of A549 cells *in vitro* and *in vivo* by inhibiting the EphA2/Akt signaling pathway

**DOI:** 10.3892/or.2024.8774

**Published:** 2024-07-10

**Authors:** Meijuan Chen, Cheng Hu, Yuanyuan Guo, Rilei Jiang, Huimin Jiang, Yu Zhou, Haian Fu, Mianhua Wu, Xu Zhang

Oncol Rep 40: 1339–1347, 2018; DOI: 10.3892/or.2018.6531

Subsequently to the publication of the above article, an interested reader drew to the authors' attention that, for the scratch-wound assay experiments shown in Fig. 3C, two images appeared to overlap [specifically, the ‘0 h / Control’ and 0 h / OP-B (5 μmol/l) data panels], albeit with different magnification and after a 180° rotation.

The authors have examined their original data, and realize that an inadvertent error was made in assembling the images in the figure; specifically, the images of 5 and 10 μmol/l OP-B treatment for 0 h were both misused. The corrected version of Fig. 3, showing all the correct data for Fig. 3C, is shown on the next page. Note that these errors did not affect the overall conclusions reported in the paper. All the authors agree with the publication of this corrigendum, and are grateful to the Editor of *Oncology Reports* for allowing them the opportunity to publish this. They also apologize to the readership for any inconvenience caused.

## Figures and Tables

**Figure 3.**
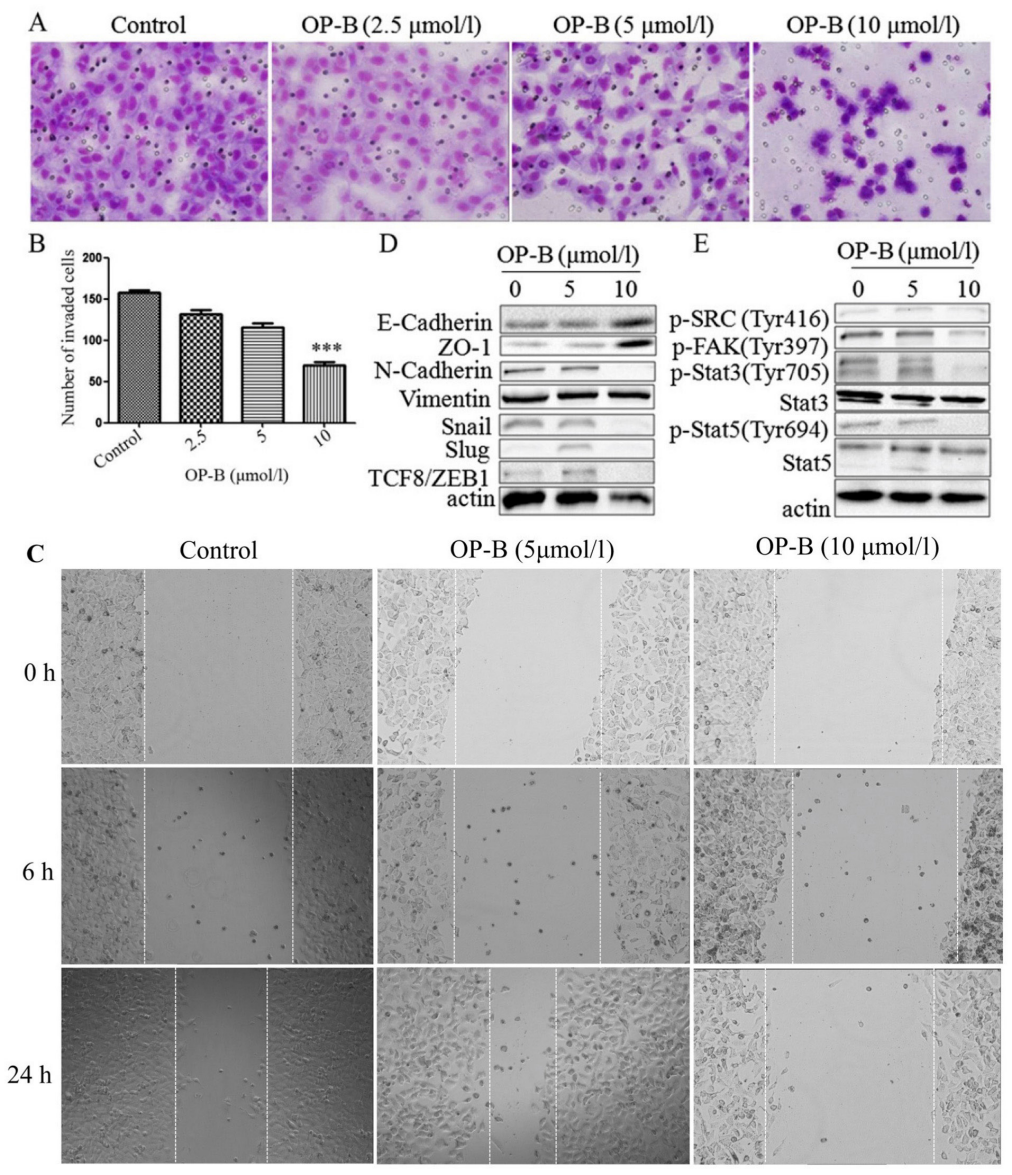
OP-B inhibits the invasion and migration of A549 cells *in vitro* and *in vivo*. (A) Transwell migration and invasion assays were performed to examine cell migration and invasion in A549 cells. Representative images of migrated or invaded cells are displayed (magnification, ×200). (B) Columns indicate the mean ± SD of triplicate experiments (***P<0.001, independent Student's t-test). (C) Wound healing assays were used to investigate the motility of A549 cells treated with OP-B, and representative images are shown (magnification, ×40). (D and E) A549 cells were treated with or without 10 µmol/l OP-B for 24 h, and then, the expression levels of vimentin, N-cadherin, E-cadherin, ZO-1, Snail, Slug, TCF8/ZEB1, p-SRC (Tyr416), p-FAK (Tyr397), p-Stat3 (Tyr705), Stat3, p-Stat5 (Tyr694) and Stat5 were detected by western blotting. β-actin was used as a loading control. The experiment was repeated three times and yielded similar results. OP-B, Ophiopogonin B. OP-B inhibits the invasion and migration of A549 cells *in vitro* and *in vivo*. (F) OP-B inhibits the lung metastasis of A549 cells *in vivo*. Representative images of H&E-stained metastatic lung nodules. OP-B, Ophiopogonin B.

